# Differences in pharmacodynamic responses to rocuronium in normal or injured orbicularis oris are associated with expression of acetylcholine receptor subunits

**DOI:** 10.1038/s41598-017-03549-4

**Published:** 2017-06-12

**Authors:** Yong Huang, Yian Xing, Hong Wang, Lianhua Chen, Shitong Li

**Affiliations:** Department of Anesthesiology, Shanghai General Hospital, Shanghai Jiao Tong University School of Medicine, Shanghai, China

## Abstract

Previous research has indicated that differences in sensitivities to muscle relaxants exist between facial nerve- and somatic nerve-innervated muscles. Here, we report that the 50% inhibitory concentration (IC50) values for rocuronium were significantly larger in the normal orbicularis oris than those in the gastrocnemius. Increased IC50 values and reduced twitch tension were observed after facial nerve injury. The normal orbicularis oris had a smaller muscle fiber cross-sectional area (CSA) and a larger ratio of endplate surface area (ESA) to muscle fiber CSA (ESA/CSA), but no difference was found in the density of nicotinic acetylcholine receptor (nAChR) subunits on endplates between normal orbicularis oris and gastrocnemius. Expression of the nAChR α1, β1, δ, ε, and γ subunits increased significantly on the postsynaptic membranes of endplates and extra-junctional muscle membranes after facial nerve injury. Our results suggest that facial nerve-innervated muscle was less sensitive than somatic nerve-innervated muscle, and the mechanisms underlying this result may be related to muscle fiber CSA and the ESA/CSA ratio, but not to the density of nAChR subunits on endplates. Facial nerve injury caused the resistance to neuromuscular blockers and reduced twitch tension, which was related to qualitative, quantitative, and locational changes in nAChR subunits.

## Introduction

Evoked electromyography (EEMG) is widely used during neurosurgery, otorhinolaryngology, and skull base surgery to detect the location of the facial nerve, which can reduce the incidence of iatrogenic facial nerve injury^1^. The EEMG response relies on adequate neuromuscular signal transmission between the facial nerve and the muscles it innervate; however, the signal is precisely blocked by non-depolarizing muscle relaxants (NDMRs), which are frequently used during general anesthesia. In our previous clinical study, partial neuromuscular blockade (PNMB) was used to achieve sufficient muscle relaxation to ensure body immobility, while maintaining sufficient facial nerve neuromuscular conduction to allow the use of EEMG^2^. These results are in accordance with other reports^3, 4^ showing that muscles innervated by the facial and somatic nerves might have different sensitivities to muscle relaxants.

In addition, the majority of patients will likely have different degrees of facial nerve injury before surgery, and the EEMG response may have already decreased due to inflammation, trauma or tumor compression^5^. Our previous study also showed that the EEMG response is affected more by facial nerve injury of greater severity^6^. To avoid iatrogenic injury in patients with pre-existing or unchecked impaired facial nerve function, EEMG monitoring should be applied with extra caution in such patients.

The nicotinic acetylcholine receptor (nAChR) on the motor endplate (also known as the neuromuscular junction [NMJ]) is the target of NDMRs. The nAChR is upregulated as a result of burns and immobilization, and denervation may change the response to NDMRs, which are linked to quantitative or qualitative changes in the nAChR^7–9^. Skeletal muscle nAChRs are comprised of five homologous subunits (2α_1_, β_1_, γ/ε, and δ) and can be divided into adult-type nAChR (mature nAChR or ε-AChR) and fetal-type nAChR (immature nAChR or γ-AChR), which have different electrophysiological characteristics, resulting in pharmacodynamic changes in NDMRs. Thus, the different sensitivities to muscle relaxants between facial nerve- and somatic nerve-innervated muscles might be associated with quantitative or qualitative differences in nAChR subunits at the NMJ. Moreover, the different degrees and time courses of facial nerve injuries may alter pharmacodynamic responses to NDMRs, which could be related to the altered subunit formation induced by nerve injury.

This study was designed to test pharmacodynamic responses to the muscle relaxant rocuronium in muscles innervated by somatic and facial nerves with or without an injury *in vitro*. We also assessed the dynamic quantitative, qualitative, and locational changes in nAChR subunits following facial nerve injury. We aimed to provide theoretical evidence on the usage of PNMB in the patients with different degrees of latent pre-surgical facial nerve injury referring intraoperative facial nerve EEMG monitoring in general anesthesia.

## Results

### Histopathological examination of injured facial nerves

**Figure 1:**
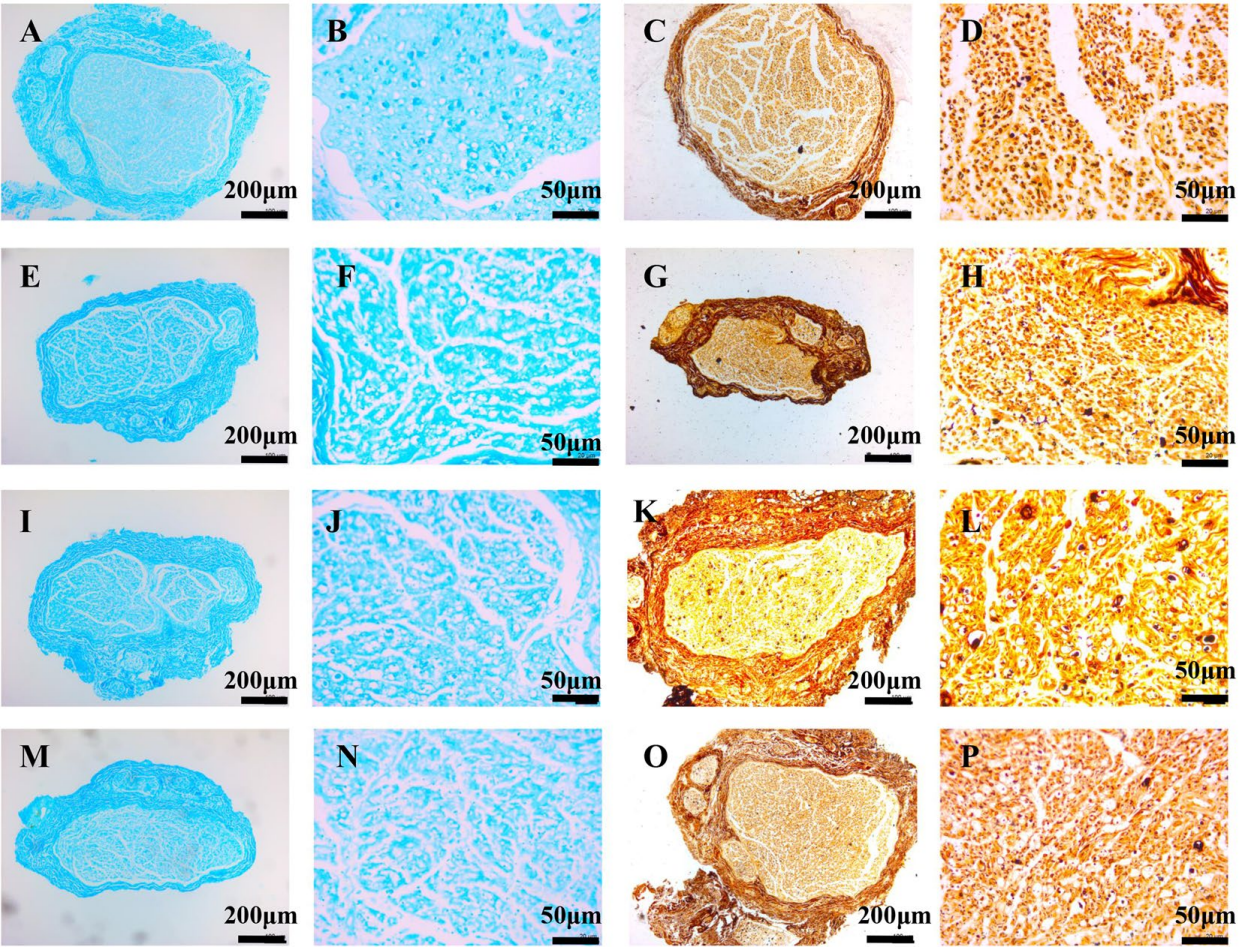
Histopathological examination of injured facial nerves on day 7 by Luxol fast bule staining (**A,B,E,F,I,J,M,N**) and Bielschowsky staining (**C,D,G,H,K,L,O,P**). Grade I injured facial nerve (A-D) had an integrated perineurium and continuous axons with well-distributed and aligned myelin sheaths in the cross-sections. Grade II injured facial nerve (**E–H**) had patchy loss of myelin sheaths with intact nerve bundles. Grade III injured facial nerve (**I–L**) had substantial thinning of myelin sheaths and blistered and vacuolar degenerative axons. Most of the myelin sheaths and axons were disintegrated in Grade IV injured facial nerve (**M–P**).

Given the severity of the facial nerve injury model, the rats in the injury group were assigned to four groups: DI, DII, DIII, and DIV (see details of group assignments in the Methods section). Nerves in the DI injury group had an approximately normal morphological appearance, with an integrated perineurium and continuous axons with well-distributed and aligned myelin sheaths in the cross-sections at all time points. The DII injury group exhibited patchy loss of myelin sheaths with intact nerve bundles on day 7; swollen axons, thickened perineurium, and obvious demyelination on day 14 but recovered to normal after day 30. The nerve bundles in the DIII injury group were intact but the intima had disintegrated with substantial thinning of myelin sheaths and blistered and vacuolar degenerative axons on day 7; the myelin sheaths had almost completely disaggregated to be almost as severe as axonal disaggregation, resulting in barely recognizable nerve bundles on day 14; some neuronal degeneration accompanied by different extents of remyelination and axon regeneration were detected on day 30, which almost recovered to normal after day 30. Most of the myelin sheaths and axons were disintegrated in the DIV group, and the nerve bundles were severely damaged or broken, but the nerve trunks were continuous through the epineurium (Fig. 1). These findings were in accordance with the characteristics of grades I–IV nerve injury described by Sunderland^10^.

### Rocuronium dose-response curves

**Figure 2:**
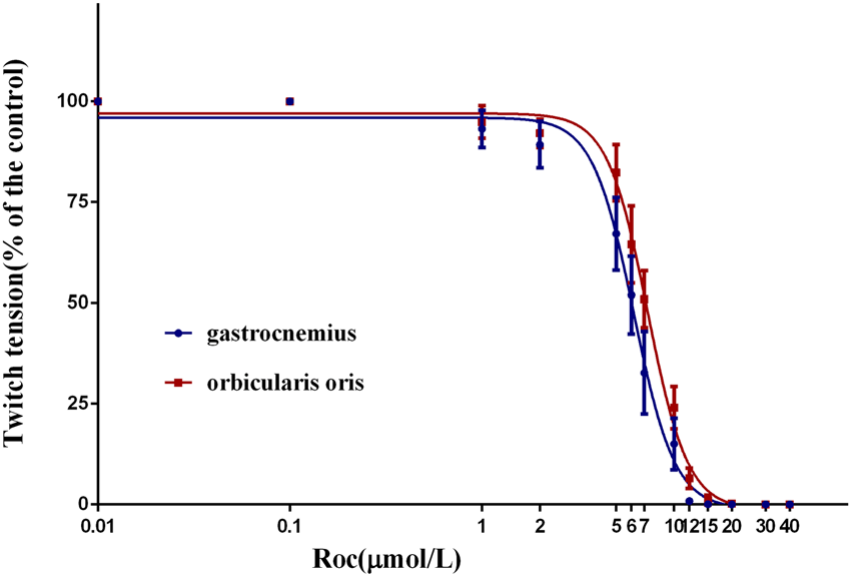
Rocuronium dose-response curves for the normal orbicularis oris and normal gastrocnemius. Values are means ± standard deviation.

Rocuronium reduced the magnitude of indirectly elicited twitch tension of the orbicularis oris and gastrocnemius dose-dependently in normal rats, (P < 0.05; Fig. 2). The IC50 value, which quantitatively indicates the position on the curve, was significantly larger for the normal orbicularis oris (7.28, 7.10–7.46) than that for the gastrocnemius (6.17, 6.00–6.35). No difference in the slope was observed between normal orbicularis oris and gastrocnemius (P < 0.05).

**Figure 3:**
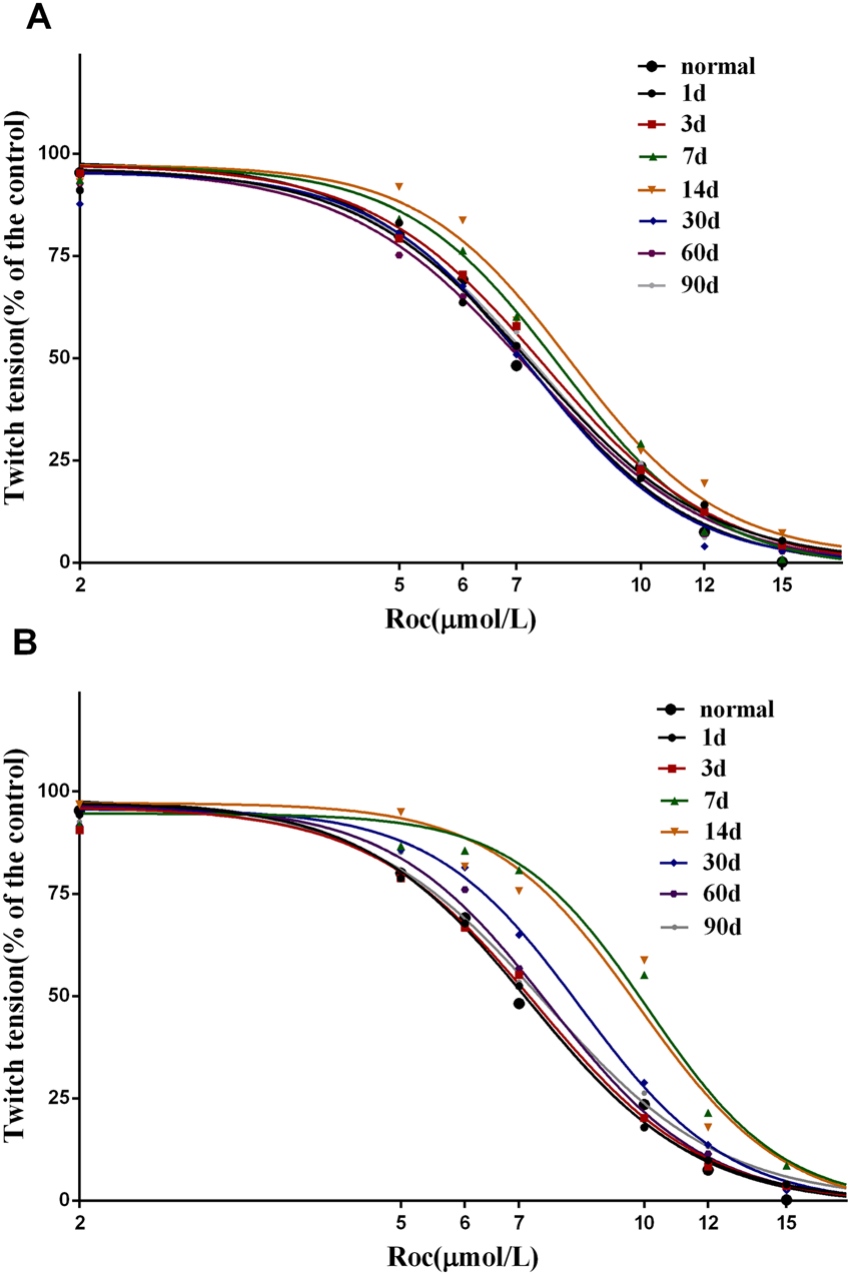
Rocuronium dose-response curves for (**A**) the DIII (grade III) injury and (**B**) DIV (grade IV) injury groups. Values are means.

No differences were observed in the IC50 values between the normal, DI, and DII injury groups at any time after facial nerve injury (P > 0.05, data not shown). The IC50 value in the DIII injury group increased significantly on day 14 compared with that in the normal group (P < 0.05; Fig. 3A; Table 1). The IC50 values in the DIV injury group increased significantly on days 7 and 14 compared with those in the normal, day 1, day 3, day 30, day 60, and day 90 groups (P < 0.05). The slope of the dose-response curve in the DIV injury group increased on day 7 compared with that in the normal, day 1, day 3, and day 90 groups (P < 0.05; Fig. 3B; Table 1). The IC50 values for the injured facial nerve-innervated orbicularis oris were positively correlated with the extent of facial nerve injury on days 7 (r = 0.69, P < 0.05) and 14 (r = 0.86, P < 0.01).

**Table 1:** IC50 values and slopes of the rocuronium dose-response curves for the normal, DIII (grade III), and DIV (grade IV) facial nerve injury groups.

	Normal	Facial nerve injury group						
		Day 1	Day 3	Day 7	Day 14	Day 30	Day 60	Day 90
		DIII						
IC50	7.28 (7.10–7.46)	7.34 (6.88–7.82)	7.59 (7.15–8.05)	7.95 (7.55–8.38)	8.16* (7.80–8.55)	7.28 (6.91–7.68)	7.26 (6.82–7.73)	7.47 (7.04–7.93)
Hillslope	4.15 ± 0.19	3.98 ± 0.43	4.01 ± 0.39	4.47 ± 0.39	4.62 ± 0.35	4.46 ± 0.44	3.83 ± 0.40	4.06 ± 0.40
		DIV						
IC50		7.21 (6.82–7.62)	7.37 (6.99–7.77)	10.11*^#▴☆Δ★^ (9.60–10.65)	9.84*^#▴☆Δ★^ (9.29–10.43)	8.33*^#^ (7.90–8.79)	7.62 (7.23–8.02)	7.54 (7.04–8.09)
Hillslope		4.26 ± 0.43	4.18 ± 0.38	5.19 ± 0.61*^#▴★^	4.78 ± 0.54	4.74 ± 0.43	4.44 ± 0.40	3.96 ± 0.45

IC50 values and slopes at the IC50 are means with 95% confidence intervals and means ± standard deviation, respectively. *P < 0.05 vs. normal; ^#^P < 0.05 vs. day 1; ^▴^P < 0.05 vs. day 3; ^☆^P < 0.05 vs. day 30; ^Δ^P < 0.05 vs^.^ day 60; ^★^P < 0.05 vs. day 90.

### Twitch tension in the facial nerve injury groups

**Figure 4:**
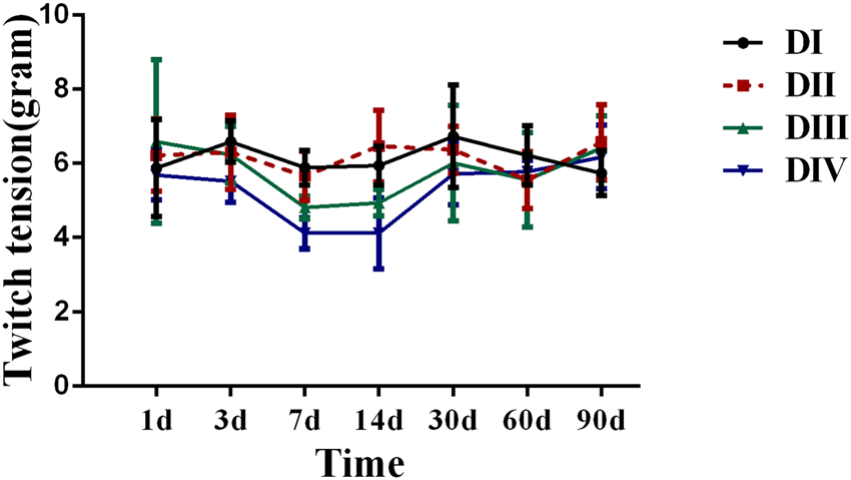
Twitch tension in the DI (grade I), DII (grade II), DIII (grade III), and DIV (grade IV) facial nerve injury groups. Values are means ± standard deviation.

The single twitch tension magnitude values in the normal, DI, and DII injury groups were not different from each other at any time (P > 0.05; data not shown). In the DIII injury group, the single twitch tension magnitude values on day 7 were significantly smaller than those in the normal, day 1, day 3, day 30, and day 90 groups; the single twitch tension magnitude values on day 14 were significantly smaller than those in the normal, day 1, day 3, and day 90 group. In the DIV injury group, the single twitch tension magnitude values in the day 7 and day 14 injury groups were significantly smaller than those in the normal, day 1, day 3, day 30, day 60, and day 90 groups (Fig. 4).

### Expression of acetylcholine receptor subunits

**Figure 5:**
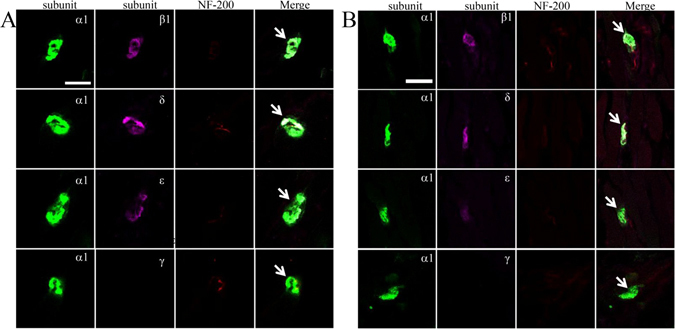
Confocal micrographs showing localization of the α1, β1, δ, ε, and γ subunits at the neuromuscular junctions (NMJs) of the (**A**) gastrocnemius and (**B**) normal orbicularis oris. Immunofluorescence staining with α-bungarotoxin (green, mark α1 subunit), β1 or δ or ε or γ subunits (pink), and neurofilament 200 (red, mark nerve). Arrows mark the NMJs. Scale bars = 30 μm.

The results of normal immunofluorescent staining are shown in Fig. 5. We used an AlexaFluor 488-conjugated α-bungarotoxin and neurofilament 200 to detect NMJs. α-Bungarotoxin is a snake venom that specifically binds to the acetylcholine receptor α1 subunit. The endplates of normal orbicularis oris and gastrocnemius were clearly visible, as ribbon-like compact and bright structures. All expression of the α, β, δ, and ε subunits was confined to the NMJ in normal orbicularis oris and gastrocnemius, whereas the γ subunit was not observed in the NMJ or muscle membranes. No differences in the density of the α, β, δ, or ε subunits were detected in the NMJ between normal orbicularis oris and gastrocnemius.

**Figure 6:**
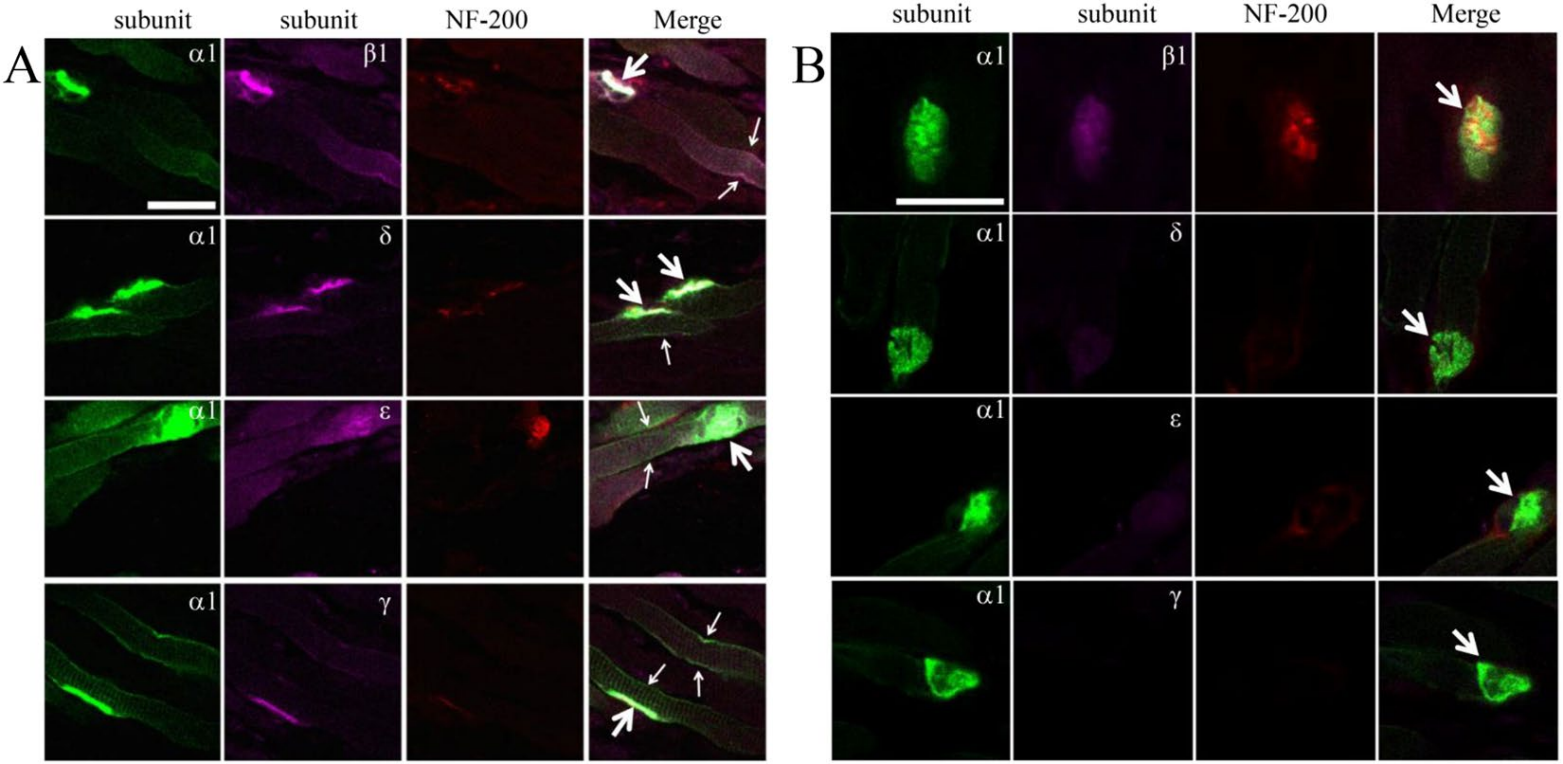
Confocal micrographs showing localization of the α1, β1, δ, ε, and γ subunits at the NMJs and muscle membrane of (**A**) DIV (grade IV) facial nerve injury of the orbicularis oris on day 7 and (**B**) DIV facial nerve injury of the orbicularis oris on day 30. Immunofluorescence staining with α-bungarotoxin (green, mark α1 subunit), β1, or δ or ε or γ subunits (pink), and neurofilament 200 (red, mark nerve). Large arrows mark the NMJ, and small arrows mark the muscle membrane. Scale bars = 30 μm.

**Figure 7:**
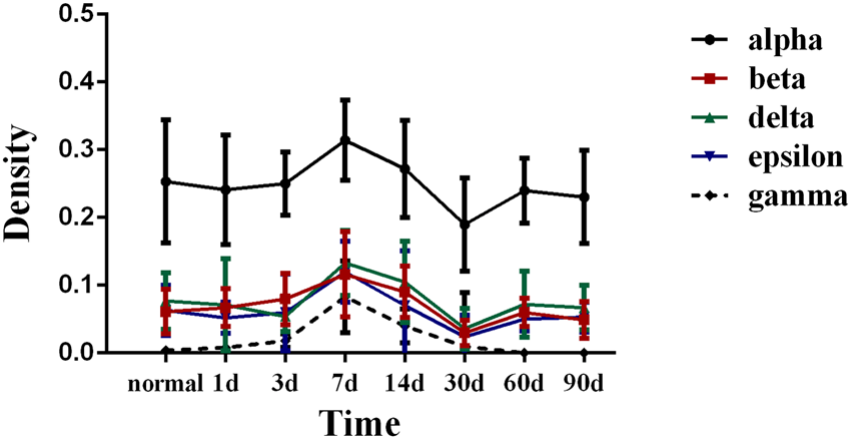
Optical density of α, β, δ, ε, and γ subunits at the NMJs in the DIV (grade IV) facial nerve injury group. All values are means ± standard deviation.

In the DIV facial nerve injury group, the α, β, δ, and ε subunits were not only significantly upregulated in the NMJ, but also linearly localized on the muscle membrane on day 7. The γ subunit also accumulated along the entire skeletal muscle membrane, including the postsynaptic membrane of the NMJ on day 7. Then, expression of the α, β, δ, ε, and γ subunits decreased. The nAChR became diffuse and dimmer on day 30, which was associated with a decrease in nAChR subunits density and an increase in endplate area (187.21 ± 41.32 μm^2^). The majority of the α, β, δ, and ε subunits accumulated on the postsynaptic membrane of the NMJ on day 30. And little γ subunits were observed on muscle membranes on day 30 (Figs 6 and 7). The nAChR became relatively normal on days 60 and 90. The changes in the DI, DII, and DIII groups were not as obvious as those in the DIV group, so their images are not shown.

### NMJ and muscle fiber morphological data

We observed α-bungarotoxin immunoreactivity at the NMJ, as well as the fiber size of the normal orbicularis oris and gastrocnemius muscle. The muscle fiber cross-sectional area (CSA) and endplate surface area (ESA) of the orbicularis oris were significantly lower than those of the gastrocnemius (P < 0.05). The orbicularis oris was composed of small fibers and a large ESA relative to muscle fiber CSA (ESA/CSA) compared with those of the gastrocnemius (Table 2).

**Table 2:** Morphological data for normal facial nerve-innervated orbicularis oris and tibial nerve-innervated gastrocnemius.

Muscle	Mean fiber cross section area (CSA, μm^2^)	Endplate surface area (ESA, μm^2^)	ESA/CSA
Orbicularis oris	301.85 ± 83.19^*^	148.39 ± 33.28^*^	0.53 ± 0.21^*^
Gastrocnemius	628.79 ± 47.15	251.82 ± 26.34	0.40 ± 0.06

The size of the ESA was measured by analyzing the circumference of the total endplate region from transverse sections. The size of the mean fiber CSA was measured from longitudinal sections in the same way. Values are means ± standard deviation. Significant differences detected by the paired *t-*test. ^*^P < 0.05 vs. gastrocnemius.

## Discussion

The IC50 values for rocuronium between the orbicularis oris and gastrocnemius were taken as measures of the muscle’s pharmacodynamic response to rocuronium. The results showed that the IC50 of the orbicularis oris was higher than that of the gastrocnemius, suggesting that the responses to rocuronium were less sensitive in the facial nerve-innervated muscles compared with those in the tibial nerve-innervated muscles, which was in accordance with our previous experimental studies^6, 11–13^. The increase in the IC50 value suggests lower affinities of the nAChR, demonstrating that the orbicularis oris was hyposensitive to rocuronium. This result strongly supports our previous clinical research^2^, in which partial neuromuscular blockade (PNMB) provided adequate conditions for EEMG monitoring and maintained the body immobility necessary for general anesthesia. Therefore, the result support the use of PNMB during surgery involving facial nerve monitoring, which has clinical significance for avoiding hemodynamic depression or delayed emergence from anesthesia in situations where deep levels of anesthesia are used instead of a muscle relaxant.

The mechanisms of the different sensitivities to NDMRs between different muscles include perfusion, temperature, endplate size, muscle fiber size, fiber composition, acetylcholine receptor, and quantal release of acetylcholine. In the *in vitro* study, the factors such as perfusion and temperature could be excluded, so we focused on the endplate size, muscle fiber size, and acetylcholine receptor subunits in the current study. We previously discovered, by using radioimmunoassay, that the density of nAChR per unit fiber CSA in the orbicularis oris is significantly higher than that in the gastrocnemius, and hypothesized that when most of the nAChR at the endplates of tibial nerve-innervated muscles are occupied by muscle relaxants the endplates of the facial nerve-innervated muscles still have adequate unoccupied receptors to retain neuromuscular transmission sufficient to obtain an EEMG recording^11^. For the consideration that we did not detect the specific nAChR location on the NMJ in our previous study, we further investigated the density of nAChR subunits on the NMJ more accurately in this study. However, no difference in the density of nAChR subunits on the NMJ was detected, but the orbicularis oris had a higher density of nAChR per unit fiber CSA on the NMJ because of larger muscle fiber CSA. However, Ibebunjo suggested that differences in acetylcholine receptor density may not account for the unequal sensitivities of normal muscles to neuromuscular drugs^3^. He concluded that the sensitivity of muscle relaxants varies directly with muscle fiber CSA and inversely with the ESA/CSA ratio^14–16^. That is, muscles with small fibers or large endplates relative to muscle fiber CSA are more sensitive to muscle relaxants, which was consistent with our studies.

Little is known about the mechanisms underlying the association between muscle sensitivity and fiber size or the ratio of endplate size to fiber size. Neuromuscular signals are transmitted if the amplitude of the endplate potential reaches a certain threshold. The endplate potential (V) is equal to the entire charge (Q) divided by capacitance (C) of the fiber (V = Q/C). The entire charge is determined by the endplate current and its duration, and each activated nAChR allows a fixed current to go through during a fixed open time. Thus, it is expected that the charge delivered to the endplate remains constant at any level of receptor occupancy. However, capacitance is proportional to the muscle fiber diameter or CSA of the fiber^17^. Small capacitance, namely small fiber size, increases the possibility that the endplate potential is near or even beyond the threshold, which leads to muscle contraction and recovery from blockade by a muscle relaxant^4, 14^. Our previous studies also showed that the amplitude of the endplate potential in the orbicularis oris is larger than that of the gastrocnemius when using rocuronium^12, 13^. Hence, small fibers tended to be less sensitive to muscle relaxants. Considering that the orbicularis oris has a smaller muscle fiber CSA and relatively larger ESA/CSA ratio, and that the density of AChR at the endplate did not differ between these two muscles, the orbicularis oris had a relatively higher quantity of nAChR per fiber. Thus, we proposed that when most nAChR on the NMJ of the gastrocnemius were saturated with muscle relaxant, the NMJ of the orbicularis oris had a sufficient number of unoccupied receptors to complete neuromuscular transmission, leading to the lower sensitivity to rocuronium. Combined with our previous findings^11^ and serial studies by Ibebunjo^3, 4, 14, 15^, we conclude that facial nerve-innervated muscle is hyposensitive to muscle relaxants than somatic nerve-innervated muscle, and the mechanisms may be related to muscle fiber CSA and the ESA/CSA ratio, but not to the density of nAChR subunits at endplates. We regarded this finding may be one of the mechanisms to explain the usage of PNMB in the surgeries referring EEMG monitoring.

In some pathological status, reduced EEMG responses could be observed after burn, immobilization, and denervation, and it is widely believed to be due to the up-regulation of nAChR. This study involved twitch tension responses as well as dynamic pharmacodynamic responses of rocuronium on orbicularis oris at different times after varying degrees of facial nerve injury. The denervation stage affects resistance to a neuromuscular blocker differently^18^. However, the effect of varying degrees of facial nerve injury on rocuronium remains unclear. In this study, resistance to rocuronium was positively correlated with severity of the nerve injury, but the IC50 did not increase in the DI and DII groups. Resistance to rocuronium was evident on day 7 in the DIII group and manifested on days 7 and 14 in the DIV group. Upregulation of junctional and extra-junctional localization of the nAChR α, β, δ, ε and γ subunits was observed during this period. Hence, the resistance was correlated with qualitative, quantitative, and locational changes in the receptor.

Only the adult-type nAChR is synthesized in mature muscle cells and is confined to the NMJ, whereas the fetal-type nAChR is re-expressed after denervation and spread throughout the muscle fiber membrane. Compared with adult-type nAChR, the fetal-type nAChR has different electrophysiological characteristics and affinity for agonists, resulting in different potencies of NDMRs on γ-nAChR and ε-nAChR^19, 20^. γ-AChR is more sensitive to ACh, as lower doses of ACh can depolarize the γ-nAChR^21, 22^. Thus, upregulation of immature nAChR would increase the amount of NDMR required to competitively block ACh^23^, and this requirement of different neuromuscular blockers varies^22^. Moreover, the increased slope of the dose-response curve on day 7 also implied altered affinity of nAChR to NDMRs, which was due to a change in the quality of the nAChR. Interestingly, immature receptors not only existed in the extra-junctional muscle membrane at the early stage after nerve injury, but also presented in the junctional muscle membrane. Whether these two distinct parts of immature receptors, which are located differently, contribute to the differential resistance to NDMRs is unclear. Beyond that, some studies have found that α7-nAChR may be upregulated in some pathological states, leading to pharmacodynamic changes in neuromuscular blockers^24, 25^.

The increase in endplate area corresponded to a decrease in nAChR α, β, δ, ε, and γ subunit density on day 30 in the DIV facial nerve injury group, suggesting that the NMJs reorganized during this period^26^. Thus, we assumed that the overall quantity of mature nAChR on the NMJ might be nearly unchanged on day 30 compared with that in the normal group, but that the few immature nAChRs on the muscle membrane were able to change the pharmacodynamic responses of orbicularis oris to NDMRs.

Remarkably, no difference in the proportion of twitch tension inhibited in muscle innervated by normal or injured facial nerves was seen at several rocuronium doses in our previous study *in vitro* ^11^. As shown above, the resistance to rocuronium was positively correlated with the severity of nerve injury. If the nerve was stretched and not seriously bruised or torn, for example in that case, the pharmacodynamic response to NDMRs barely changed. Then, we further measured the maximum extent of EEMG inhibition (EEMG%) *in vivo* in a facial nerve injury rabbit model of different severities (grades I–III), as well as the time course, to mimic the clinical situation more closely^6^. However, no differences were found in EEMG% between the normal and any grade of facial nerve injury after a single bolus administration of rocuronium. It is important to consider the differences in the methods used when comparing our present results with previous findings. We used a cumulative dose response method, in contrast to a single bolus method in the rabbit study. In addition, indirectly elicited twitch tension was obtained at over a dozen concentrations to construct the dose-response curve in contrast to maximal EEMG inhibition. Therefore, based on these findings, we conclude that although maximal EEMG inhibition did not differ between normal and injured orbicularis oris, a significant difference in the pharmacodynamic response to rocuronium was detected between normal and injured orbicularis oris.

In the present study, reduced twitch tension magnitudes of the orbicularis oris elicited by indirect facial nerve stimulations were observed on day 7 after nerve injury and persisted on day 14, which was associated with an increase in fetal-type nAChR on days 7 and 14. Muscle activity is directly modulated by nerve impulses, transmitted from motor neurons via axons to nAChR on muscle fibers, which induce muscle contraction. The mature nAChR isoform normally mediates muscle contraction, whereas the immature nAChR contributes minimally to neurotransmission^8^. The ratio of mature to immature receptors is quantitatively lower after nerve injury^18^ and, therefore, contributes less to neurotransmission. Witzemann *et al*.^27^ used epsilon-subunit gene knockout mice, which only have fetal-type nAChR on the NMJ, and found impaired neuromuscular transmission and muscle weakness. The mechanisms by which the fetal-type nAChR influence muscle properties may be related to a prolonged open channel time, which could cause Ca^2+^ overload of the junctional cytoplasm thus leading to endplate myopathy^28^. Consistent with this hypothesis, and with the proposed association between twitch tension and immature nAChR, twitch tension did not decrease after day 14 in the present study. Therefore, expression of the immature nAChR at the NMJ may have contributed to the decline in twitch tension on days 7 and 14.

In conclusion, we got two findings from the experiment. First, facial nerve-innervated muscle was hyposensitive to muscle relaxant rocuronium than somatic nerve-innervated muscle, and the underlying mechanism may be related to muscle fiber CSA and the ESA/CSA ratio, but not to the density of nAChR subunits on endplates. This provides theoretical evidence for applying a PNMB during surgeries requiring facial nerve monitoring under general anesthesia. Second, facial nerve injury caused reduced twitch tension responses and resistance to neuromuscular blocker rocuromiun in the innervated muscles, which was related to qualitative, quantitative, and locational changes in the nAChR subunits. Resistance to rocuronium was positively correlated with severity of the nerve injury; this may be the mechanism to explain the reduced EEMG responses in the situations. Thus, extra caution should be exercised when muscle relaxants are used during surgery requiring EEMG monitoring for preoperatively damaged facial nerves.

## Methods

### Animals

This study was approved by the Animal Care and Use Committee of the Shanghai Jiao Tong University. A total of 178 male Sprague–Dawley rats (weight range: 200–250 g) were obtained from the Experimental Animal Center of Shanghai General Hospital (Shanghai, China). All rats received humane care according to the Care and Use Committee of Shanghai Jiao Tong University. All experimental procedures involving animals were approved by the Animal Ethics and Use Committee of Shanghai Jiao Tong University.

### Group assignments and facial nerve injury model

The rats were divided randomly into two groups: (i) a normal group (n = 10); and (ii) a facial nerve injury group (n = 168). Given the severity of the facial nerve injury model, the rats in the injury group were assigned to four groups: DI, DII, DIII, and DIV (injury grades I–IV). Each injury group was further divided into seven subgroups containing six rats each, in which pharmacodynamic experiments were performed on days 1, 3, 7, 14, 30, 60, and 90 after facial nerve injury.

The rats were fasted before the experiments but were given free access to water and food at other times. The left-sided facial nerve injury was induced using the crush injury model, as described previously^29^. A standard crush using the same microvascular hemostatic forceps (W40340; Shanghai Medical Instruments Co, Shanghai, China) was performed for a defined amount of time to cause one of the graded injuries according to the Sunderland method^10^, in which crushing one buckle of forceps for 10 s results in a grade I injury, crushing one buckle for 60 s produces a grade II injury, crushing two buckles for 60 s produces a grade III injury, and crushing three buckles for 60 s produces a grade IV injury. Success was assessed based on damage to facial nerve function on the left side, including loss of the blink reflex, vibrissae orientation, and mouth drooping on the left side after the animals recovered from anesthesia. Nerve samples from the same segments of the injured facial nerves were obtained to check their integrity in a pathological examination. Luxol fast bule staining was used to observe changes in nerve myelin, and Bielschowsky staining was used to observe changes in axons.

### Muscle preparations and rocuronium pharmacodynamics

The rats were killed with 60 mg/kg pentobarbital injected intraperitoneally. The buccal branches of the facial nerves were exposed carefully to avoid damaging the muscles, nerves, and vessels. Firstly, the muscles was carefully freed of surrounding tissues, keeping the blood supply remained intact, then the nerve was sectioned and finally the vessels were cut. The left facial nerve-innervated orbicularis oris muscle affiliated with the buccal branch, and the left tibial nerve-innervated gastrocnemius muscle affiliated with the nerve, were dissected into strips (1 × 2 cm). Each isolated strip was mounted vertically in a tissue chamber filled with Krebs solution, maintained at 37 °C, and bubbled with 95% oxygen and 5% CO_2_. The strips were suspended with an L hook on one side and connected to a force displacement transducer on the other side (ALC-M System for Isolated Tissue-Organ Research; Shanghai Alcott Biotech, Shanghai, China; 40 mL in volume). The nerves were positioned on wire bipolar platinum electrodes for indirect stimulation.

Indirect electrical stimulation-evoked twitch tension was recorded with an MPA Multiple Channel Biological Signal Analysis System (provided by Shanghai General Hospital, School of Medicine, Shanghai Jiao Tong University). The proximal end of the nerve attached to the strips was stimulated with a single supramaximal train of rectangular pulses (intensity, 15 V; duration, 0.05 ms; frequency, 0.1 Hz,). The pulse was repeated three times at 5-s intervals, and mean twitch tension amplitude was calculated. Baseline twitch tension amplitude was recorded before rocuronium (Organon, Oss, The Netherlands) was added. Rocuronium was applied to the preparation at concentrations of 0.01, 0.1, 0.5, 1, 2, 5, 6, 7, 10, 12, 15, 20, 30, and 40 μmol/L. The drug concentrations were determined by adding freshly prepared solutions with calibrated micropipettes to modified Krebs solution (40 mL) in the tissue chamber. After the drug effect stabilized for a minimum of 10 min, single-twitch tension was determined. Data were accepted when the effects of rocuronium were completely reversible by washing with Kreb’s solution. Indirectly elicited twitch tension in the same preparation with no neuromuscular blocker was defined as the control value. Dose-response curves were constructed and the 50% inhibitory concentration (IC50) values were obtained.

### Immunofluorescence staining

The muscles were fixed in cold acetone for 10 min. Then, the muscles were rinsed with PBS and placed in a 20% sucrose solution for 1 h, followed by a 30% sucrose solution overnight at 4 °C, and finally frozen in liquid nitrogen. Serial 30-μm thick longitudinal or transverse sections were cut using a cryostat at about −25 °C. The samples were blocked with 10% normal donkey serum for 30 min at room temperature and then incubated with primary antibodies in 10% normal donkey serum overnight at 4 °C. After washing in PBS, the samples were incubated with secondary antibodies and AlexaFluor α-bungarotoxin (1:1,000, B13422; Life Technologies, Carlsbad, CA, USA). The primary antibodies for immunofluorescence were goat anti-beta subunit (1:100, sc-1448; Santa Cruz Biotechnology, Santa Cruz, CA, USA), mouse anti-delta subunit (1:200, ab2804; Abcam, Cambridge, UK), goat anti-epsilon subunit (1:100, sc-1455; Santa Cruz Biotechnology), goat anti-gamma subunit (1:100, sc-1453; Santa Cruz Biotechnology), and rabbit anti-neurofilament 200 (1:200, N4142; Sigma-Aldrich, St. Louis, MO, USA). The secondary antibodies were AlexaFluor 594 donkey anti-goat (1:1,000, A11058; Life Technologies), AlexaFluor 594 donkey anti-mouse (1:1,000; A21203, Life Technologies), and AlexaFluor 647 donkey anti-rabbit (1:1,000; A31573; Life Technologies). After a final wash with PBS, the samples were embedded and viewed under a confocal laser microscope (TCS SP8; Leica, Heidelberg, Germany). The settings of the confocal microscope, including laser power, gain, magnification, thickness of the stacks, and other factors were the same for all experiments. Sections treated without primary antibodies were used as controls.

### Statistical analysis

The competition analysis data (IC50 and slope at the IC50) were determined from a four-variable logistic sigmoidal dose-response model, fitted to the dose-response curves using Prism 6 software (GraphPad Software, Inc., San Diego, CA, USA). The IC50 values are expressed as means with 95% confidence intervals, whereas other data are presented as means with standard deviations and were analyzed with SPSS statistical software (ver. 22.0; SPSS Inc., Chicago, IL, USA). Image analysis measurements were made using Image-Pro Plus (ver. 5.1; Media Cybernetics, Silver Spring, MD, USA) by a blind observer. At least ten transverse sections from each muscle sample were studied for the quantified data of ESA. For each section, 5 to 10 NMJs were randomly selected in the mid-portion of the muscle. The size of the ESA was measured by analyzing the circumference of the total endplate region. The size of the mean fiber CSA was measured from longitudinal sections in the same way. A minimum of 50 fibers or NMJs from non-consecutive sections were analyzed for each muscle. The density of the subunits was calculated after subtracting background fluorescence. Significant differences were assessed using one-way repeated-measures analysis of variance, followed by analysis of variance with the least-significant difference test. The paired *t*-test was used to compare between normal orbicularis oris and gastrocnemius muscle data. The correlation analysis for the IC50 values and the extent of facial nerve injury was conducted using Spearman’s test. A *P*-value < 0.05 was considered significant.

### Data availability

All data are available upon request, please contact Yong Huang (email address: 1953354139@qq.com).
